# Zero-dose children in Turkey: regional comparison of pooled data for the period 1990 to 2018

**DOI:** 10.1186/s12879-022-07416-0

**Published:** 2022-05-02

**Authors:** Mehmet Ali Eryurt, Siddika Songül Yalçin

**Affiliations:** 1 Institute of Population Studies, Hacettepe University, Ankara, Turkey; 2 Department of Pediatrics, Faculty of Medicine, Hacettepe University, Ankara, Turkey

**Keywords:** Zero-dose children, Unvaccinated, Turkey, Demographic and Health Survey, Regional inequality

## Abstract

**Background:**

Immunization plays a vital role in child health and survival. Zero-dose children are coming increasingly into focus as part of the global Immunization Agenda 2030. Although the percentage of zero-dose children has decreased in Turkey over time, regional/socioeconomic inequalities persist. This study aims to analyze the trend in zero-dose children and the factors associated with this problem in Turkey in light of regional inequalities.

**Methods:**

Six data sets (1993, 1998, 2003, 2008, 2013, and 2018) were pooled from the last six Turkey Demographic and Health Surveys (TDHSs). The vaccination module for children aged 12–35 months and variables related to household characteristics, socio-economic, cultural characteristics of parents, bio-demographic/health-related factors were taken from the DHS data. Binary logistic regression analyses were carried out by taking into account the complex sample design of surveys for Turkey in general, the East region, and other regions.

**Results:**

Significant progress has been made in reducing the number of zero-dose children in Turkey over the last three decades, as it has dropped from 3.2 to 0.9%. The results of multivariate analyses revealed that survey year, household wealth, the mother’s level of education, payment of bride price, mother’s native language, place of delivery, and the number of antenatal care visits are associated with zero-dose children. Factors associated with zero-dose children also differ between the East region, and other regions.

**Conclusion:**

Public health programs targeting uneducated parents, poor households, lack of social security, Kurdish-speaking mothers, older mothers and those without antenatal care should be implemented to promote childhood immunization.

## Introduction

Immunization is recognized globally as one of the most cost-effective measures to improve child survival. Despite the dramatic increase in childhood immunization rates, the 90% target set by the World Health Organization (WHO) has not yet been achieved. The 2030 Sustainable Development Goals (SDGs) can only be reached when there are no more zero-dose children [1]. Children who receive the first dose are much more likely to complete their vaccination schedules. Zero-dose children are those who have not received any vaccine, not even a single dose of vaccine has been given. Therefore, identifying the characteristics of zero-dose children and addressing barriers to immunization will likely improve equity in immunization coverage. A previous global estimate for zero-dose vaccination based on 241 representative household surveys in 96 countries up to 2007 was 10% [2]. A recent study determined that 7.7% of children were zero-dose in national surveys of 92 low- and middle-income countries (45 DHS and 47 MICS countries) from 2010 to 2020 [3]. Several national studies have been conducted to evaluate vaccination coverage [4–7]. However, these studies generally investigated only one period. The factors associated with zero-dose children might change over time within countries. To encourage and facilitate immunization programmes, there is a need for long-term studies that evaluate and monitor changes within countries at regional levels [8]. The DHS database contains a rich set of variables related to the characteristics of the household where the child lives, socio-economic and cultural characteristics of the parents and bio-demographic/health related factors and questions regarding childhood vaccination. Therefore, several factors associated with zero-dose children can be easily investigated using the DHS data.

In Turkey, routine vaccines, including BCG, Hepatitis B, oral polio vaccine, pentavalent vaccine (DTaP, IPV, Haemophilus influenzae type b), Streptococcus pneumonia, and measles-mumps-rubella, varicella and hepatitis A are included in the National Immunization Program and given free of charge to all children throughout the country. The Ministry of Health has tried to achieve a target of at least 90% vaccination coverage at the national and regional levels through public health facilities [9, 10]. The catch-up schedule for both zero-dose and partially vaccinated children has been defined by the Ministry of Health [10]. The percentage of zero-dose children declined from 3.1% in TDHS-1993 to 0.9% in TDHS-2018 throughout Turkey [11, 12]. The country was divided into five regions (West, South, Central, North, and East) according to socio-economic and geographical characteristics. Regional disparities not only indicate geographical disparities, but also social, economic and cultural differences [13]. The West region is the most industrialized and the most socially and economically developed region of Turkey. The South, North and Central regions follow the West region. The East region is the least developed region in the country with low percentages of maternal education and a high birth rate [12, 14–17]. While childhood immunization coverage has increased since 1990, inequities among regions have remained [11, 12, 14–17]. Some studies have evaluated some provinces, and there is one cross-sectional sampling in Turkey [18–20]. However, studies that include a detailed assessment of zero-dose vaccination on a regional basis are lacking. Therefore, this study aimed to provide data regarding the magnitude of the problem with zero-dose children and the trend for the last three decades and to describe predictors associated with this problem, for Turkey overall and separately for the regions. The results of the study may provide an important factual foundation for formulating effective regional vaccination policies in the future.

## Methods

### Data source

Data were obtained from the last six Turkey Demographic and Health Surveys (TDHSs) conducted by the Hacettepe University Institute of Population Studies in 1993, 1998, 2003, 2008, 2013 and 2018. All surveys are nationally representative household surveys, and a weighted, multi-stage and stratified cluster sampling was performed on all surveys and to a large extent similar questionnaires were used [11, 12, 14–17]. These similarities made it possible to pool data sets and to obtain a large data set. Face-to-face structured interviews were conducted with women 15 to 49 years of age.

Data for children who were alive at the time of the TDHS fieldwork and who were born in the 3 years preceding the survey were included in the study. To obtain vaccination data for each eligible child, mothers were asked whether they had a vaccination card for the child, and if so, to show the card to the interviewer. The vaccination dates and doses were copied from the card to the questionnaire. If a vaccination card was not available for the child, then the mother was asked a series of questions in order to determine the vaccination status of the child.

We restricted the study sample to children aged 12–35 months and living with their mothers during the survey. If a mother had more than one child in this age range, we selected the younger child for inclusion in our analysis. A child was not included in the analysis when no information on vaccination status was provided (missing cases), but if the mother did not remember whether the child had ever been vaccinated, the child was treated as a zero-dose child. Finally, a total of 8198 mother–child pairs were eligible and included.

### Variables

The dependent variable in the study is vaccination status, which is a binary variable with a value of “1” if the child is zero-dose and a value of “0” if the child is vaccinated. In addition to the survey year variable, which measures the period effect, variables related to household characteristics, parental characteristics, cultural characteristics and bio-demographic/health related characteristics were included in the study as independent variables. Factors related to household characteristics included environmental factors such as place of residence (urban/rural), region and household wealth. Parental characteristics consisted of the mother’s level of education, the father’s level of education (No education/primary incomplete, primary or secondary and higher), parental working status (at least one having social security, neither having social security) and health insurance. Cultural variables included bride payment, arranged marriage and mother’s native language (Turkish, Kurdish but also speaking Turkish, Kurdish, not speaking Turkish or other). Bio-demographic and health-related factors such as the mother’s age at birth (< 20, 20–34, or ≥ 35 years), the sex of the child, the age of the child, parity and birth interval (1st child, 2nd child, interval < 24 months; 2nd child, interval ≥ 24 months; ≥ 3rd child, interval < 24 months; 3rd child, interval ≥ 24 months), number of antenatal care visits (none, 1–3 or ≥ 4), place of delivery (home or healthcare facility), and previous tetanus vaccination of the mother (yes or no). Variables such as the father’s level of education, parental health insurance, and arranged marriage were not included in multivariate analyses so as not to cause multicollinearity. Previous tetanus vaccination of the mother is only included in the descriptive analysis as the data is present only in the 1993 and 1998 TDHS surveys.

### Sampling designs

While the sample designs and questionnaires of the six surveys were the same, sample sizes differed. Hence, the number of women interviewed and the number of children whose vaccination information was collected were different. In order to avoid possible biases in the analyses stemming from the different number of observations in different surveys, weighting factors obtained with the Eq. 1

1 $${1\div({\text{a}} \times {\text{n}}_{{\text{c}}} /{\text{n}}_{{\text{T}}})}$$

were used [21], where a is the number of surveys, n_c_ is the number of respondents for survey c, and n_T_ is the total number of respondents for all surveys.

### Statistical analysis

Analyses were carried out using the IBM SPSS 23.0 complex samples module, taking into consideration the sample design of the survey, which was selected through a multi-stage, stratified cluster-sampling approach, and taking into account the simple non-random sample selection of the data set. In the complex samples procedure cluster, strata and weight variables in the related TDHS surveys data were all accounted for. We calculated zero-dose prevalence and its 95% confidence interval (95% CI) for each sub-population group taking into account the complex survey design. Binary logistic regression analysis was used as the technique to identify the factors associated with zero-dose children. CSLOGISTICS command was used to perform complex samples logistic regression. In the binary logistic regression, the Exp (B) (odds ratio) values obtained from the analysis indicate the probability of the failure of the dependent variable in relation to the likelihood of its fulfillment. The Exp (B) value of an independent variable shows in which direction and to what extent it affects the likelihood of the fulfillment of the dependent variable. Values of less than 1 indicate that it reduces the likelihood of fulfillment, whereas values greater than 1 indicate that it increases the likelihood. Descriptive, univariate, and multivariate analyzes were conducted to measure the impact of independent variables on zero-dose children. The analyses were made at three levels: Turkey overall (national), the East region and other regions (West, South, Central and North regions). As the differentiation is predominantly between the East region and other regions and the number of observations in the West, South, Central and North regions is not sufficient, these four regions were evaluated and analyzed together.

### Ethics

This study was a secondary data analysis of DHS data, which was approved by the institutional ethical review board of Hacettepe University, Turkey. All respondents undergo an informed consent process for participation in the surveys. Additional ethical approval was not needed for this study as it used publicly available data from from the Institute of Population Studies.

## Results

Out of 7693 children (weighted sample), each survey dataset contributed 16–17% of the total pooled data. One fourth of the weighted sample was from the East Region and 30% from rural areas. Demographic, parental and child characteristics are provided in Table 1. Table 1 clearly reveals the disadvantages faced by the East region. There are large differences in the distribution of characteristics in the East region (Region 5) versus Turkey overall (national) and other regions (Region 1–4, i.e. the west, south, central and north regions). For instance, while 45% of the children in the East region are living in the poorest households, this percentage decreases to 22% nationally and to 15% in other regions. Similarly, 53 percent of mothers had no education or never finished primary school, this percentage decreases to 23% nationally and to 13% in other regions.

**Table 1:** Percentage and frequency distribution of the characteristics of survey population

	National		Regions: 1–4		Region: 5: East	
	n*	n** (%)	n*	n** (%)	n*	n** (%)
Total	8198	7693 (100)	5332	5725 (100)	2866	1968 (100)
Zero-dose children						
Yes	185	154 (2.0)	53	53 (0.9)	132	10 (5.1)
No	8013	7539 (98.0)	5279	5672 (99.1)	2734	1867 (94.9)
Survey year						
1993	1346	1330 (17.3)	1086	1006 (17.6)	260	324 (16.5)
1998	1308	1312 (17.0)	925	986 (17.2)	383	326 (16.6)
2003	1669	1268 (16.5)	953	924 (16.1)	716	343 (17.4)
2008	1490	1284 (16.7)	869	966 (16.9)	621	318 (16.2)
2013	1374	1266 (16.5)	852	938 (16.4)	522	328 (16.7)
2018	1011	1232 (16.0)	647	905 (15.8)	364	329 (16.7)
Residence						
Urban	5473	5322 (69.2)	3767	4228 (73.9)	1706	1095 (55.6)
Rural	2725	2370 (30.8)	1565	1496 (26.1)	1160	873 (44.4)
Region						
1. West	1711	2578 (33.5)				
2. South	1233	1055 (13.7)				
3. Central	1450	1577 (20.5)				
4. North	938	515 (6.7)				
5. East	2866	1968 (25.6)				
Household wealth						
Poorest	2168	1753 (22.8)	893	866 (15.1)	1275	887 (45.1)
Poor	1873	1700 (22.1)	1159	1200 (21.0)	714	500 (25.4)
Middle	1618	1586 (20.6)	1203	1293 (22.6)	415	292 (14.8)
Rich	1371	1349 (17.5)	1081	1176 (20.5)	290	172 (8.7)
Richest	1168	1305 (17.0)	996	1188 (20.8)	172	116 (5.9)
Mother’s level of education						
No education/Prim. incomp	2189	1807 (23.5)	744	770 (13.4)	1445	1037 (52.7)
Primary	3595	3409 (44.3)	2668	2805 (49.0)	927	604 (30.7)
Secondary and higher	2414	2477 (32.2)	1920	2150 (37.6)	494	328 (16.7)
Father’s level of education						
No education/Prim. incomp	718	620 (8.1)	237	268 (4.7)	481	351 (17.8)
Primary	4155	3947 (51.3)	2701	2907 (50.8)	1454	1039 (52.8)
Secondary and higher	3286	3093 (40.2)	2375	2528 (44.2)	911	565 (28.7)
DK/Missing	39	34 (0.4)	19	21 (0.4)	20	12 (0.6)
Parental working status						
At least one having social security	4438	4440 (57.7)	3400	3735 (65.2)	1038	705 (35.8)
Neither having social security	3664	3161 (41.1)	1871	1920 (33.5)	1793	1241 (63.1)
Missing	96	92 (1.2)	61	70 (1.2)	35	22 (1.1)
Health insurance						
No	2652	2435 (31.7)	1617	1660 (29.0)	1035	774 (39.3)
Yes	5520	5231 (68.0)	3701	4049 (70.7)	1819	1182 (60.1)
DK/Missing	26	26 (0.3)	14	15 (0.3)	12	12 (0.6)
Bride payment						
No	6183	6004 (78.0)	4648	4971 (86.8)	1535	1034 (52.5)
Yes	2015	1689 (22.0)	684	755 (13.2)	1331	934 (47.5)
Arranged marriage						
By themselves	3585	3420 (44.5)	2432	2683 (46.9)	1153	737 (37.4)
By families	4233	3898 (50.7)	2604	2721 (47.5)	1629	1178 (59.9)
Escaped/Abducted/Other	380	375 (4.9)	296	322 (5.6)	84	53 (2.7)
Mother’s native language						
Turkish	5571	5539 (72.0)	4689	4972 (86.8)	882	567 (28.8)
Kurdish, but also speaking Turkish	1763	1441 (18.7)	426	524 (9.2)	1337	917 (46.6)
Kurdish, not speaking Turkish	471	355 (4.6)	22	22 (0.4)	449	334 (17.0)
Other, unknown	393	358 (4.7)	195	207 (3.6)	198	151 (7.7)
Mother’s age at the birth (year)						
< 20	900	852 (11.1)	599	633 (11.1)	301	218 (11.1)
20–34	6524	6120 (79.6)	437	485 (8.5)	337	234 (11.9)
≥ 35	774	720 (9.4)	4296	4605 (80.4)	2228	1516 (77.0)
Gender						
Male	4228	3961 (51.5)	2751	2933 (51.2)	1477	1028 (52.2)
Female	3970	3732 (48.5)	2581	2792 (48.8)	1389	940 (47.8)
Child’s age (months)						
12–23	4139	3876 (50.4)	2683	2873 (50.2)	1456	1003 (51.0)
24–35	4059	3817 (49.6)	2649	2852 (49.8)	1410	965 (49.0)
Parity and birth interval						
1st child	2745	2679 (34.8)	2053	2211 (38.6)	692	468 (23.8)
2nd child, interval < 24 months	557	518 (6.7)	348	368 (6.4)	209	149 (7.6)
2nd child, interval ≥ 24 months	1716	1711 (22.2)	1325	1459 (25.5)	391	252 (12.8)
≥ 3rd child, interval < 24 months	738	624 (8.1)	283	302 (5.3)	455	322 (16.4)
≥ 3rd child, interval ≥ 24 months	2442	2161 (28.1)	1323	1383 (24.2)	1119	777 (39.5)
Number of antenatal care visits						
No antenatal visits	1460	1243 (16.2)	656	625 (10.9)	804	619 (31.5)
1–3	1633	1395 (18.1)	971	963 (16.8)	662	432 (22.0)
≥ 4 or more	4565	4570 (59.4)	3474	3854 (67.3)	1091	716 (36.4)
DK/missing	540	485 (6.3)	231	282 (4.9)	309	202 (10.3)
Place of delivery						
Hospital	6722	6441 (83.7)	4702	5124 (89.5)	2020	1314 (66.8)
Home	1476	1251 (16.3)	630	600 (10.5)	846	655 (33.3)
Maternal tetanos vaccination during pregnancy^#^						
No	1332	1414 (53.5)	885	946 (47.5)	447	469 (72.0)
Yes	1322	1228 (46.5)	1126	1046 (52.5)	196	182 (28.0)

^*^Unweighted count, **Weighted count

^#^Data were present in 1993 and 1998 survey

**Fig. 1:**
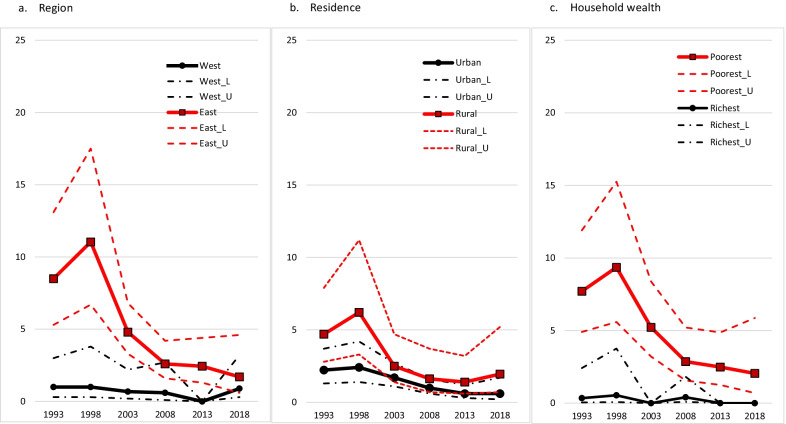
Trend of the proportion and 95% confidence interval (CI) of zero-dose children according to region (**a**), residence (**b**) and household wealth (**c**). (L:lower CI, U:upper CI)

**Fig. 2:**
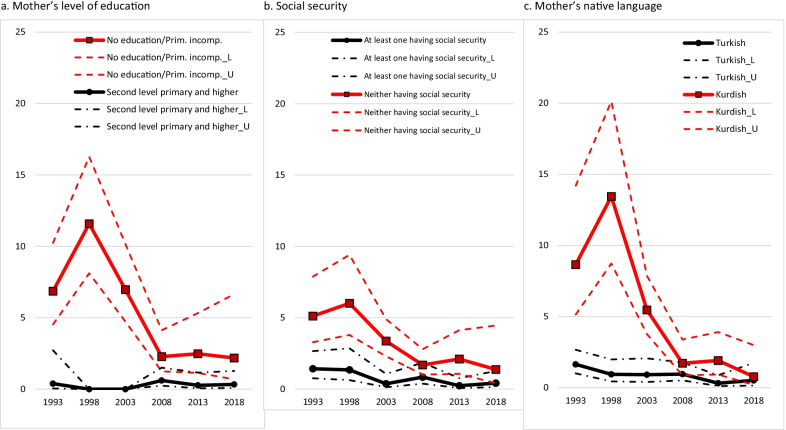
Trend of the proportion and 95% confidence interval (CI) of zero-dose children according to maternal education (**a**), parental working status with social security (**b**) and mother’s native language (**c**). (L:lower CI, U:upper CI)

Pooling the six TDHS data sets created an important opportunity to examine the trend of zero-dose children in sub-population groups over the last 30 years. Figures 1 and 2 show that the differences between sub-population groups have decreased over time, and there has been convergence in the period from 1993–2018. The graphs presented in Fig. 1 depict a decrease in differences between urban/rural settlements, regions, and the welfare status of the household where the child lives especially in the 2000s. Similarly, differences related to the mother’s level of education, whether parents held a job with social security, and the native language of the mother lessened over time (Fig. 2). The convergence that occurred is primarily due to the strengthening of the healthcare system and more accessible health services.

According to the 2018 TDHS results, although the percentage of zero-dose children varies significantly by sub-population group, regional/socio-economic inequalities persist. From 1993 to 2018, the percentage of zero-dose children declined from 3.2 to 0.9 across Turkey (Table 2). Although the decline in the East region is much more striking (8.5% to 1.7%) than in other regions, the percentage of zero-dose children in the East region was still significantly higher in 2018 than in other regions (Fig. 1).

**Table 2:** The distribution of zero-dose according to characteristics of mother–child pairs in Regions: 1–4 and Region: 5

	National			Regions 1–4*			Region 5: East		
	n	%	95% CI	n	%	95% CI	n	%	95% CI
Total	154	2.0	1.6–2.5	53	0.9	0.7–1.3	101	5.1	4.0–6.6
Survey year									
1993	42	3.2	2.2–4.6	15	1.5	0.9–2.4	27	8.5	5.3–13.1
1998	50	3.8	2.5–5.8	14	1.4	0.7–2.8	36	11.0	6.7–17.5
2003	26	2.0	1.4–2.9	9	1.0	0.4–2.2	16	4.8	3.3–6.8
2008	15	1.2	0.8–1.8	7	0.7	0.3–1.5	8	2.6	1.6–4.2
2013	10	0.8	0.4–1.3	2	0.2	0.0–0.7	8	2.4	1.3–4.4
2018	11	0.9	0.4–1.9	6	0.6	0.2–1.8	6	1.7	0.6–4.6
Residence									
Urban	72	1.4	1.1–1.7	28	0.7	0.4–1.0	44	4.0	3.0–5.4
Rural	81	3.4	2.5–4.7	24	1.6	1.0–2.5	57	6.5	4.4–9.5
Region									
1. West	18	0.7	0.4–1.2						
2. South	13	1.2	0.7–2.3						
3. Central	15	1.0	0.6–1.7						
4. North	6	1.3	0.6–2.8						
5. East	102	5.1	4.0–6.6						
Household wealth									
Poorest	88	5.0	3.9–6.4	23	2.7	1.7–4.2	64	7.3	5.4–9.6
Poor	37	2.2	1.6–3.1	12	1.0	0.5–2.0	25	5.0	3.4–7.4
Middle	20	1.3	0.8–2.0	11	0.9	0.5–1.6	9	3.0	1.5–6.0
Rich	6	0.4	0.2–0.9	4	0.4	0.1–1.0	1	0.8	0.2–3.1
Richest	3	0.2	0.1–0.6	1	0.1	0.0–0.5	1	1.2	0.2–6.0
Mother’s level of education									
No education/Prim. incomp	107	5.9	4.8–7.3	30	3.9	2.5–5.9	78	7.5	5.9–9.5
Primary	39	1.1	0.8–1.7	17	0.6	0.4–1.0	22	3.7	2.1–6.2
Secondary and higher	7	0.3	0.2–0.6	6	0.3	0.1–0.6	2	0.5	0.2–1.3
Father’s level of education									
No education/Prim. incomp	40	6.4	4.6–8.7	7	2.7	1.1–6.3	32	9.2	6.7–12.6
Primary	96	2.4	1.9–3.1	37	1.3	0.9–1.8	58	5.6	4.0–7.8
Secondary and higher	18	0.6	0.4–0.9	8	0.3	0.2–0.7	9	1.6	0.9–2.9
DK/Missing	1	2.9	0.7–11.2				1	7.9	1.9–27.9
Parental working status									
At least one having social security	32	0.7	0.5–1.0	21	0.6	0.3–0.9	11	1.6	1.0–2.7
Neither having social security	117	3.7	2.9–4.7	29	1.5	1.0–2.3	88	7.1	5.4–9.2
Missing	5	5.4	1.9–14.0	3	3.9	0.8–16.6	2	9.9	2.8–29.2
Health insurance									
Yes	96	3.9	3.0–5.1	27	1.7	1.1–2.5	68	8.8	6.6–11.8
No	55	1.0	0.8–1.4	24	0.6	0.4–0.9	30	2.5	1.9–3.5
DK/Missing	3	12.4	4.4–30.4	1	4.5	0.6–27.0	3	22.3	6.5–54.4
Bride payment									
No	56	0.9	0.7–1.2	33	0.7	0.4–1.0	23	2.2	1.5–3.3
Yes	98	5.8	4.5–7.5	20	2.6	1.6–4.3	78	8.4	6.3–11.1
Arranged marriage									
By themselves	38	1.1	0.8–1.5	16	0.6	0.3–1.0	22	3.0	2.0–4.4
By families	106	2.7	2.1–3.5	32	1.2	0.8–1.7	75	6.4	4.7–8.6
Escaped/Abducted/Other	9	2.5	1.3–4.6	5	1.7	0.7–4.2	4	7.4	3.3–15.6
Mother’s native language									
Turkish	50	0.9	0.7–1.2	42	0.9	0.6–1.2	8	1.4	0.7–2.7
Kurdish, but also speaking Turkish	47	3.3	2.3–4.6	7	1.4	0.6–3.2	40	4.4	3.1–6.2
Kurdish, not speaking Turkish	46	13.0	10.0–16.8	2	7.5	1.8–26.2	45	13.4	10.2–17.3
Other, unknown	10	2.8	1.4–5.6	2	0.7	0.1–5.0	9	5.7	2.7–11.5
Mother’s age at the birth (year)									
< 20	16	1.9	1.1–3.3	1	0.2	0.1–1.0	15	6.7	3.8–11.6
20–34	111	1.8	1.4–2.3	10	2.1	1.1–4.3	16	6.8	4.3–10.5
≥ 35	26	3.7	2.5–5.4	41	0.9	0.6–1.2	71	4.7	3.5–6.2
Gender									
Male	75	1.9	1.4–2.5	23	0.8	0.5–1.2	52	5.1	3.6–7.0
Female	79	2.1	1.6–2.7	30	1.1	0.7–1.6	49	5.2	3.9–7.0
Child’s age (months)									
12–23	75	1.9	1.5–2.5	23	0.8	0.5–1.2	52	5.2	3.8–7.0
24–35	79	2.1	1.6–2.7	30	1.0	0.7–1.6	49	5.1	3.6–7.2
Parity and birth interval									
1st child	30	1.1	0.7–1.7	8	0.4	0.2–0.8	22	4.6	2.8–7.5
2nd child, interval < 24 months	15	2.9	1.6–5.2	5	1.5	0.5–4.0	9	6.3	3.1–12.7
2nd child, interval ≥ 24 months	17	1.0	0.6–1.6	11	0.8	0.4–1.3	6	2.3	1.1–4.8
≥ 3rd child, interval < 24 months	29	4.7	3.2–6.9	5	1.8	0.7–4.6	24	7.4	4.9–11.1
≥ 3rd child, interval ≥ 24 months	63	2.9	2.2–3.8	22	1.6	1.0–2.6	40	5.2	3.9–6.9
Number of antenatal care visits									
No antenatal visits	92	7.4	5.7–9.7	20	3.2	1.9–5.2	73	11.7	8.8–15.6
< 4	25	1.8	1.2–2.7	15	1.6	0.9–2.8	10	2.2	1.2–4.2
≥ 4 or more	27	0.6	0.4–0.8	14	0.4	0.2–0.6	13	1.8	1.0–3.0
DK/missing	10	2.0	1.1–3.7	3	1.2	0.4–3.5	6	3.1	1.4–6.6
Place of delivery									
Hospital	64	1.0	0.8–1.3	26	0.5	0.3–0.8	38	2.9	2.1–4.0
Home	89	7.1	5.4–9.3	26	4.4	2.8–6.8	64	9.6	6.9–13.3
Maternal tetanos vaccination during pregnancy**									
No	81	5.7	4.2–7.8	21	2.2	1.3–3.6	61	12.9	9.3–17.7
Yes	11	0.9	0.5–1.6	8	0.8	0.4–1.6	3	1.5	0.5–4.7

^*^Regions 1–4 includes West, South, Central and North regions

^**^data were present in 1993 and 1998 survey

CI: confidence interval

Univariate analyses showed that from 1993 to 2018, the odds of being a zero-dose child decreased, with statistically significant results starting in 2008 (Table 3). Living in rural settlements, living in the East region, living in poor households, having a mother with a low level of education, having a father with a low level of education, neither parent working a job with social security, lack of health insurance, payment of bride price during the wedding of the parents, having parents with an arranged marriage, having a mother whose native language is not Turkish, having a mother whose age at birth is over 35 years old, having a short birth interval and high birth order, lack of antenatal care and being born at home increased the likelihood of being a zero-dose child. The child’s age and sex did not influence the likelihood of being a zero-dose child. Although the odds ratios differ depending on the separate analyses performed for the East region and other regions (regions 1–4), it is possible to make the same evaluations for the national results as for the regional results. The direction of the relationships does not change, i.e. living in poor households, having a mother with a low level of education, etc. increases the odds of being a zero-dose child in the regional analysis as well (Table 3).

**Table 3:** Factors associated with zero-dose children in Turkey, in Regions 1–4**, and Region 5 of Turkey, pooled data from TDHS-1993 to TDHS-2018, complex sample binary logistic regression***

	National				Regions 1–4				Region 5: East			
	Univariate model		Multivariate model		Univariate model		Multivariate model		Univariate model		Multivariate model	
	OR	95% CI	AOR	95% CI	OR	95%CI	AOR	95% CI	OR	95%CI	AOR	95% CI
Survey year												
1998 vs. 1993	1.20	0.66–2.19	1.32	0.75–2.31	0.96	0.41–2.23	1.36	0.52–3.55	1.34	0.66–2.71	1.20	0.60–2.41
2003 vs. 1993	0.62	0.36–1.08	0.71	0.40–1.23	0.66	0.26–1.68	1.14	0.41–3.20	0.54*	0.29–1.00	0.54*	0.28–1.02
2008 vs. 1993	0.36*	0.20–0.66	0.65	0.32–1.34	0.48	0.19–1.20	1.46	0.43–4.97	0.29*	0.14–0.58	0.42*	0.17–1.03
2013 vs. 1993	0.23*	0.11–0.46	0.57	0.25–1.31	0.12*	0.03–0.53	0.39	0.07–2.12	0.27*	0.12–0.59	0.56	0.20–1.63
2018 vs. 1993	0.28*	0.12–0.64	0.75	0.29–1.95	0.42	0.13–1.34	1.48	0.36–6.14	0.19*	0.06–0.59	0.41	0.12–1.40
Residence												
Rural vs. urban	2.57*	1.70–3.89	0.81	0.54–1.20	2.47	1.32–4.63	0.91	0.42–1.97	1.65	1.00–2.74	0.74	0.47–1.17
Region												
West vs. east	0.13*	0.07–0.24	0.60*	0.29–1.21								
South vs. east	0.23*	0.12–0.47	0.63*	0.30–1.29								
Central vs. east	0.18*	0.10–0.33	0.59*	0.30–1.16								
North vs. east	0.23*	0.10–0.55	0.61*	0.25–1.50								
Household wealth												
Poor vs. poorest	0.43*	0.29–0.63	0.67*	0.46–0.98	0.37	0.17–0.82	0.68	0.29–1.60	0.68	0.45–1.03	0.70	0.45–1.09
Middle vs. poorest	0.24*	0.14–0.42	0.62	0.34–1.16	0.32	0.15–0.70	0.76	0.30–1.95	0.39*	0.18–0.85	0.60	0.24–1.50
Rich vs. poorest	0.08*	0.04–0.19	0.33*	0.14–0.81	0.14	0.05–0.39	0.40	0.13–1.25	0.10*	0.04–0.24	0.32	0.07–1.39
Richest vs. poorest	0.04*	0.01–0.12	0.23*	0.07–0.78	0.04	0.01–0.18	0.16*	0.03–0.81	0.15*	0.03–0.81	0.56	0.12–2.61
Mother’s level of education												
Primary vs. ≤ Prim. incomp	0.18*	0.12–0.27	0.59	0.34–1.02	0.15	0.08–0.29	0.30*	0.14–0.62	0.47*	0.27–0.80	1.04	0.57–1.89
≥ Secondary vs. ≤ Prim. incomp	0.05*	0.02–0.10	0.37*	0.16–0.86	0.07	0.03–0.17	0.28*	0.09–0.85	0.06*	0.02–0.17	0.30*	0.09–1.02
Father’s level of education												
Primary vs. ≤ Prim. incomp	0.36*	0.25–0.54			0.47*	0.47–0.47			0.59*	0.38–0.91		
≥ Secondary vs. ≤ Prim. incomp	0.08*	0.05–0.15			0.12*	0.12–0.12			0.16*	0.09–0.32		
Parental working status												
Neither having social security vs at least one having social security	5.27*	3.50–7.95	1.27	0.83–1.95	2.76*	1.51–5.04	0.80	0.40–1.60	4.65	2.68–8.07	1.82	0.98–3.38
Parental health insurance												
Yes vs. No	0.26*	0.18–0.37			0.36*	0.20–0.64			0.27*	0.17–0.42		
Bride payment												
Yes vs. No	6.61*	4.49–9.73	1.91*	1.25–2.91	4.11*	2.24–7.54	1.69	0.85–3.36	4.03*	2.42–6.70	2.01*	1.20–3.38
Arranged marriage												
By families vs. by themselves	2.51*	1.66–3.79			2.00*	1.01–3.97			2.18*	1.33–3.60		
Escaped/Abducted vs. by themselves	2.27*	1.10–4.69			2.91	0.96–8.84			2.55*	1.00–6.53		
Mother’s native language												
Kurdish. but also speaking Turkish vs. Turkish	3.71*	2.36–5.83	0.96	0.56–1.67	1.60	0.64–3.99	0.51	0.19–1.40	3.30*	1.52–7.19	1.84	0.79–4.27
Kurdish. not speaking Turkish vs. Turkish	16.45*	10.75–25.18	1.60	0.88–2.90	9.42*	2.11–42.00	1.21	0.21–6.88	11.17*	5.22–23.91	3.64*	1.47–8.98
Other. unknown vs. Turkish	3.17*	1.45–6.94	1.04	0.44–2.44	0.86	0.12–6.28	0.46	0.07–2.99	4.34*	1.54–12.25	2.64	0.78–9.00
Mother’s age at the birth												
< 20 vs. 20–34 year	1.05	0.60–1.81	0.73	0.41–1.29	0.26	0.06–1.13	0.24	0.05–1.08	1.48	0.82–2.66	0.87	0.43–1.75
≥ 35 vs. 20–34 year	2.05*	1.32–3.19	1.25	0.73–2.16	2.46*	1.14–5.34	1.45	0.65–3.24	1.49	0.90–2.47	1.01	0.50–2.01
Gender												
Male vs. female	0.90	0.64–1.25	0.88	0.62–1.24	0.74	0.40–1.36	0.76	0.41–1.44	0.96	0.66–1.41	0.91	0.60–1.39
Child’s age												
24–35 vs. 12–23 months	1.07	0.76–1.52	1.02	0.72–1.46	1.32	0.74–2.33	1.14	0.63–2.04	0.98	0.63–1.53	0.94	0.60–1.48
Birth order and interval												
2nd child, interval < 24 months vs. 1st child	2.65*	1.37–5.15	1.34	0.68–2.63	4.12*	1.14–14.92	1.83	0.50–6.76	1.40	0.65–3.02	1.33	0.60–2.95
2nd child, interval ≥ 24 months vs. 1st child	0.90	0.48–1.69	0.87	0.46–1.64	2.17	0.84–5.62	1.64	0.56–4.86	0.48	0.19–1.23	0.49	0.19–1.25
≥ 3rd child, interval < 24 months vs. 1st child	4.43*	2.45–7.99	0.79	0.38–1.64	5.11*	1.47–17.72	1.11	0.29–4.18	1.66	0.84–3.26	0.64	0.27–1.50
≥ 3rd child, interval ≥ 24 months vs. 1st child	2.69*	1.69–4.29	0.74	0.40–1.37	4.58*	1.82–11.52	1.20	0.38–3.86	1.13	0.69–1.87	0.56	0.28–1.11
Number of antenatal care visits												
Less than 4 vs. none	0.23*	0.14–0.38	0.51*	0.30–0.89	0.49	0.23–1.03	0.96	0.43–2.11	0.17*	0.08–0.35	0.29*	0.13–0.62
4 or more vs. none	0.07*	0.05–0.12	0.45*	0.24–0.82	0.11*	0.05–0.23	0.52	0.21–1.34	0.13*	0.07–0.26	0.49*	0.24–1.01
Place of delivery												
Home vs. hospital	7.69*	5.23–11.32	1.70*	1.04–2.78	8.89*	4.88–16.18	3.54*	1.76–7.12	3.65*	2.20–6.04	1.01	0.55–1.87

^*^Statistically significant

^**^Regions 1–4 includes West, South, Central and North regions

^***^Missing values are not shown but were controlled for.

The results of multivariate analyses revealed that, even if not statistically significant, the odds of being a zero-dose child decreased significantly from 1993 to 2018 period. Regarding variables related to household characteristics, the place of residence does not influence the likelihood of being a zero-dose child in any of the three analyses performed for Turkey overall, the East region and other regions. The region variable, which is included only in the overall Turkey analysis, had a statistically significant effect. Compared to the East region, the odds are approximately one-fourth of the East region in each of the other regions. Household wealth is among the most influential variables in all three analyses. The odds of being a zero-dose child decreases as household wealth increases (Table 3).

With regard to parental characteristics, having a mother with at least a secondary school education decreases the probability of being a zero-dose child significantly. If neither parent is working a job with social security, the odds of being a zero-dose child increases**.** This variable has a greater impact in the East region than it does in the other regions even if the results are marginally insignificant (Table 3).

In terms of cultural characteristics, payment of a bride price and mother’s native language were included in the multivariate analysis. Payment of a bride price increases the odds of being a zero-dose child in Turkey overall and in the East region. The mother’s native language is another influential cultural variable. If the mother’s native language is not Turkish, the odds of being a zero-dose child increases, especially if the mother’s native language is Kurdish, and not speaking Turkish increases the odds 3.6 times compared to those whose mother’s native language is Turkish. Mother’s native language variable produces statistically significant results in the analyses of the East region but not for Turkey overall and other regions (Table 3).

Bio-demographic, health-related variables, such as mother’s age at birth, child’s age, child’s sex, parity and birth interval are not statistically significant in any of the three analyses. On the other hand, the number of antenatal care visits and place of delivery has a statistically significant impact on the odds of being a zero-dose child in Turkey overall. Having received at least one antenatal care reduces the likelihood of being a zero-dose child significantly. The decrease is more pronounced in the East region. Having at least one antenatal care visit seems to be very important in terms of integration into the health system in the East region. Being born at home increases the likelihood of being a zero-dose child significantly compared to being born in a healthcare institution. The influence of the place of delivery is more remarkable in the analysis in other regions. Results are not statistically significant in the analysis of the East region (Table 3).

## Discussion

Significant progress has been made in reducing the number of zero-dose children in Turkey over the last three decades, from 3.2% (95% CI 2.2–4.6%) in 1993 to 0.9 (95% CI 0.4–1.9) in 2018. In the period from 1990 to 2018, the differences between sub-population groups have decreased over time and convergence has been observed. However, inequalities between the East region and other regions remain. Turkey was analyzed by dividing it into five regions in terms of socio-demographic characteristics. The percentage of zero-dose children in the East region, which is the least developed region of the country, is also considerably higher than in other regions. While the percentage of zero-dose children in other regions (Region 1–4) is 0.9% (95% CI 0.7–1.3), it increases to 5.1% (95% CI 4.0–6.6) in the East region (Region 5). A similar trend is observed when the relationship between economic development and zero-dose children is evaluated according to the income level of the countries. Likewise, zero-dose prevalence was reported to be 11.1% (10.4–11.8%) in low-income countries, 7.0% (6.7–7.3%) in lower-income countries and 5.2% (4.6–7.7%) in higher-income countries [3].

Due to the notable regional inequalities, when analyzing factors associated with zero-dose children, separate analyses were carried out for Turkey overall, the East region, and other regions. Like previous studies, the probability of being a zero-dose child declined as household wealth increased [22–24]. While household wealth has a statistically significant effect in Turkey overall and in other regions, no statistically significant effect was found in the East region, where the percentage of children living in poor households is higher than in other regions. Seventy percent of the children live in the poorest or poor households. According to the univariate analysis results, the likelihood of being a zero-dose child decreases as household welfare increases in the East region. However, in the multivariate analysis, in which variables such as parental job status were added, the effect became statistically insignificant.

Maternal education was a significant predictor of zero-dose children in all three analyses. The increase in level of education was associated with a decrease in the percentage of zero-dose children. This finding is in line with previous studies [5, 22, 23, 25, 26]. This may be attributed to the fact that educated parents have a greater understanding of the value of preventive health measures and immunization than uneducated parents do. When viewed from the perspective of regional disparities, the results of the analysis revealed some differences. While a mother having a primary school education has a significant impact compared to women with no education in the other regions, a statistically significant difference emerges only if the mother has a secondary school or higher education in the East region. This situation can be interpreted as women only being able to make a difference in the traditional, patriarchal social structure of the Eastern region if they are more educated.

Similarly, while the custom of paying a bride price was associated with zero-dose children Turkey and in the East region, the absence of a statistically significant effect in other regions may be associated with the traditional and patriarchal values that dominant in the East region. Nearly half of marriages involved payment of the bride price and more than half of the marriage in the East region were arranged. These two factors might diminish the mother’s ability to act autonomously [27]. This suggests that the decision of whether or not to vaccinate is made by family elders [28, 29].

Another important variable associated with being zero-dose children is the mother’s native language. This variable only had a statistically significant effect in the East region, where the native language of 63.6% of the children is Kurdish. While 46.6% can speak Turkish, 17% cannot speak Turkish. In the East region, children whose native language is Kurdish and who do not speak Turkish are 3.64 times more likely to be zero-dose children than those whose native language is Turkish. This indicates that there is a language barrier for some women when it comes to accessing and using the healthcare system in the East.

Among the health-related factors in the study, the variables of place of delivery and number of antenatal care visits had a statistically significant effect on being a zero-dose child. The number of antenatal care visits had significant effect in the Eastern region, the place of delivery variable in other regions, and both variables in Turkey overall.

Mothers who delivered in a healthcare facility were less likely to have zero-dose children compared to mothers who gave birth at home. This finding is supported by previous studies [22, 23, 30]. A possible explanation is the educations provided by healthcare professionals about the value of immunization. Two hypotheses can be envisaged in this regard. The first is that those who gave birth in a hospital may receive information about vaccination from health personnel, or second that they rely more on positive science. The fact that there is a negative relationship between hospital births and being a zero-dose child in regions other than the East supports the second hypothesis.

Like previous studies [22, 23, 26, 30], children born to mothers who received no antenatal care during pregnancy were more likely to be zero-dose in the multivariate analysis. This situation, which is especially prominent in the East, might be due to anticipatory guidance given during antenatal care. Furthermore, according to the results of the descriptive analysis, a negative association between zero-dose children and maternal tetanus vaccination during pregnancy in the East supports this hypothesis. Especially in the East region, the mother’s contact with the health system seems important in terms of subsequent vaccination of the children.

## Strengths and limitations

Pooling the data increased the number of included cases and the reliability of estimates. This paper is the first to present coverage estimates for zero-dose children in a standardized way using six nationally representative sets of Turkey data from 1990 to 2018. TDHS survey information on vaccination status was collected from parents for all children born in the five years preceding the survey and was based on parental recall and vaccine card records. There is, therefore, a recall and ascertainment bias. However, to avoid the memory factor, the data presented here were restricted to children who were 12–35 months and were alive at the time of survey fieldwork. On the other hand, DHS data are population-based and cover all regions of the country, so the results can be generalized. Although eligible women response rates have decreased slightly over time (TDHS-1993 95%; TDHS-2018 81%), response rates are still at high levels in Turkey [11, 12]. The high response rates mitigate doubts regarding the generalizability of the results. This is the first study to make a detailed assessment of zero-dose vaccination on a regional basis and the changes over three decades.

## Conclusion

A high percentage of zero-dose children can negatively affect the health not only of individuals but the public as well. This situation becomes even more critical when it comes to infectious diseases. Vaccination is the responsibility of parents and the state, and it is children’s right. This study attempted to reveal the challenging issues and need for vaccines on a regional basis.

This study demonstrated that the variables of region, household welfare, mother’s level of education, payment of bride price, number of antenatal care visits and place of delivery have statistically significant effects on the risk of not being vaccinated across Turkey. While the variables of survey year, mother’s level of education, payment of bride price, mother’s native language and number of antenatal care visits had a statistically significant effect in the East, significant effects of household wealth, mother’s level of education and place of delivery were found in other regions. Therefore, public health programs targeting uneducated parents, poor households, those without social security, Kurdish-speaking mothers older mothers, and those without antenatal care should be implemented to promote childhood immunization. Promoting birth at healthcare facilities in the West, South, North, and Central regions and antenatal care visits in the East region can improve immunization coverage. It is hoped that the conclusions reached by the study will help guide policy-making processes.

## Data Availability

The data of this study are available from the Hacettepe University Institute of Population Studies.
